# The psychology of learning with generative AI in foreign language education: building a theory of metacognition, trust, and human–AI co-regulation through a systematic review

**DOI:** 10.3389/fpsyg.2026.1974520

**Published:** 2026-10-08

**Authors:** Xuming Shang

**Affiliations:** School of Humanities, Xinyu University, Xinyu, China

**Keywords:** foreign language education, generative artificial intelligence, human–AI co-regulation, metacognition, trust

## Abstract

Generative artificial intelligence (GenAI) is reshaping foreign language education, yet the psychological processes underlying effective learner–AI interaction remain fragmented. This theory-building systematic review synthesizes empirical evidence on metacognition, trust, and human–AI co-regulation in GenAI-supported foreign language learning. Following PRISMA 2020 procedures, web of science and Scopus were searched for studies published from 2022 to June 2026. After screening, 51 empirical studies were included. The findings show that metacognition shapes learners’ planning, monitoring, regulation, and reflection; trust influences how learners accept, verify, modify, or reject AI-generated information and feedback; and human–AI co-regulation involves the dynamic distribution of cognitive and regulatory activity between learners and GenAI within teacher-mediated instructional contexts. AI literacy and learner agency further support critical evaluation, strategic AI use, and retention of human decision-making responsibility. Based on these findings, the review develops the metacognition–trust–co-regulation (MTC) framework, which conceptualizes effective GenAI-assisted learning as an iterative process in which metacognitive regulation informs trust, trust calibrates reliance, and reliance shapes human–AI co-regulation. The framework positions GenAI as a cognitive partner whose educational value depends on critically informed, metacognitively regulated, and humanly governed use.

## 1 Introduction

### 1.1 Generative AI in foreign language education: from technological assistance to cognitive partnership

The integration of artificial intelligence into foreign language education has evolved from programmed computer-assisted language learning (CALL) toward increasingly adaptive and interactive systems. Whereas earlier technologies primarily provided linguistic input, practice, and automated feedback, large language models (LLMs) can generate context-sensitive language, explain linguistic choices, reformulate learner texts, respond to follow-up questions, and sustain extended interaction. The rapid diffusion of ChatGPT has accelerated this transformation, with GenAI increasingly used for brainstorming, drafting, revision, translation, feedback, research, and conversational practice (Abdelhalim, 2024; Cheng et al., 2025; Abano and Estremera, 2026).

More importantly, GenAI changes the learner–technology relationship. Rather than operating within a predefined instructional structure, learners can formulate prompts, request clarification, compare alternatives, and accept, modify, or reject AI-generated suggestions. Learning consequently shifts from relatively passive information retrieval toward interactive cognitive partnership, in which learners must continuously monitor AI output, evaluate its relevance and accuracy, and determine how cognitive work should be distributed between themselves and the system. Emerging research shows that metacognitive awareness, self-regulation, prompt formulation, and trust influence how learners engage with GenAI (Abdelhalim, 2024; Cheng et al., 2025; Liu et al., 2026; Wang et al., 2026).

This transformation has also generated a rapidly expanding review literature addressing AI applications in writing, speaking, listening, reading, vocabulary, grammar, feedback, assessment, and academic integrity (Abano and Estremera, 2026; Aljohani, 2026; Amoush and Alhosban, 2025). Although these reviews document GenAI applications, benefits, and risks, they provide less explanation of how and why learners learn with GenAI. Recent work has begun to address this gap. EL Hosayny et al., for example, proposed an interactive model of metacognitive regulation in ChatGPT-assisted writing (El Hosayny et al., 2026), while Varghese and Sharma synthesized research on AI and human metacognition more broadly (Varghese and Sharma, 2025). These studies highlight the growing importance of understanding psychological regulation in AI-mediated learning, but an integrated account spanning metacognition, trust, and human–AI co-regulation—understood here as the coordination of cognitive and regulatory activity between learners and GenAI—remains limited (Zakaria et al., 2025).

The resulting theoretical gap concerns the integration of these emerging perspectives. Effective GenAI-mediated learning therefore requires learners to regulate not only their own learning but also their interaction with AI, including how they formulate requests, evaluate AI outputs, and decide when and how to use AI assistance. The present review therefore shifts the focus from what GenAI can do to how learners learn with GenAI, centering the analysis on three interconnected mechanisms—metacognition, trust, and human–AI co-regulation. This perspective provides the foundation for the theory-building systematic review and the proposed Metacognition–Trust–Co-Regulation (MTC) Framework.

### 1.2 Theoretical gap, objectives, and research questions

The preceding discussion identifies a central theoretical gap in research on generative AI (GenAI) in foreign language education. Existing reviews have documented GenAI applications across language skills, writing, speaking, feedback, and assessment, as well as concerns about accuracy, ethics, academic integrity, and over-reliance. However, psychological constructs such as metacognition, trust, self-regulation, agency, autonomy, and AI literacy remain largely fragmented across individual studies. Consequently, the field still lacks an integrated explanation of how these processes interact to shape learning during learner–AI engagement.

This gap becomes increasingly consequential as GenAI enables reciprocal interaction, allowing learners to formulate prompts, evaluate and verify responses, seek clarification, and accept, modify, or reject AI suggestions. Recent conceptual work has positioned AI as a co-regulator that participates in learners’ regulatory cycles and reshapes self-regulation (Agustin, 2026). However, an empirically grounded account of how metacognition, trust, and cognitive activity interact in GenAI-mediated foreign language learning remains limited. The present review therefore examines these three interconnected mechanisms, while treating AI literacy and learner agency as enabling capacities that shape their operation.

Building on this conceptualization, the primary objective of this theory-building systematic review is to synthesize empirical evidence on the psychological processes underlying GenAI-mediated foreign language learning and integrate their relationships into a coherent theoretical framework. More specifically, the review seeks to (1) map the psychological constructs investigated in existing research; (2) examine the roles of metacognition, trust, and self-regulation in GenAI-supported learning; (3) investigate how learners evaluate, negotiate, and regulate AI-generated information and feedback; and (4) integrate these findings into a theoretical account of how learners coordinate cognition and action with GenAI.

These objectives lead to five research questions:

*RQ1*. What psychological processes and constructs have been investigated in empirical research on GenAI-supported foreign language learning?

*RQ2*. How does metacognition shape learners’ engagement with, monitoring of, and regulation of GenAI during foreign language learning?

*RQ3*. How do learners develop and calibrate trust in GenAI, and how does trust influence their acceptance, evaluation, verification, modification, or rejection of AI-generated information and feedback?

*RQ4*. How do learners and GenAI systems participate in human–AI co-regulatory processes during foreign language learning, and what roles do self-regulation, agency, autonomy, AI literacy, prompt literacy, and feedback literacy play in these processes?

*RQ5*. How can the relationships among metacognition, trust, and human–AI co-regulation be integrated into a theoretical framework explaining how and under what conditions learners learn effectively with GenAI in foreign language education?

Accordingly, RQ1–RQ4 establish the empirical basis for the review, while RQ5 integrates these findings into the proposed Metacognition–Trust–Co-Regulation (MTC) Framework, explaining how and under what psychological and interactional conditions learning with GenAI becomes productive.

## 2 Theoretical background

Generative AI increasingly participates in cognitive and regulatory activity across learning tasks. This development builds on a longer history of research on the relationship between AI and human metacognition, which Varghese traces across several decades (Varghese and Sharma, 2025). More recent work has conceptualized AI as a co-regulator within self-regulated learning and examined metacognitive regulation in ChatGPT-assisted writing (El Hosayny et al., 2026; Agustin, 2026). Together, these strands of research provide a basis for examining how learners monitor, evaluate, and regulate their interaction with generative AI. The following sections review the theoretical foundations and conceptual boundaries of metacognition, trust, human–AI co-regulation, AI literacy, and learner agency relevant to the present review.

### 2.1 Learning with AI as cognitive partnership

The concept of cognitive partnership views technology not simply as a delivery mechanism but as part of the cognitive environment in which learning occurs. From a distributed-cognition perspective, cognitive activity can extend across learners, technological tools, representations, and other elements of the learning environment. In GenAI-mediated learning, AI can therefore contribute linguistic and cognitive resources by generating alternatives, explanations, feedback, and representations that learners incorporate into ongoing problem solving (Abdelhalim, 2024; Hsu, 2025).

This relationship is better understood as augmentation rather than replacement. Learners may delegate selected generative or routine functions to AI while retaining responsibility for goals, evaluation, and decision-making. Research indicates that the consequences of GenAI use depend partly on which aspects of a task learners delegate to AI and whether they subsequently engage with and evaluate the generated material (Abdelhalim, 2024; Lai, 2026). Research on structured human–AI collaboration likewise shows that AI can function as a scaffold for writing and critical thinking while preserving learner agency (Alyasin and Shah, 2026; Alshehri et al., 2026). Thus, the educational significance of GenAI depends not only on what the system can produce but also on how learners coordinate their own cognitive activity with AI resources and determine what cognitive work should be delegated or retained (Hsu, 2025; Lai, 2026; Alshehri et al., 2026).

### 2.2 Metacognition

Metacognition was initially conceptualized by Flavell as knowledge and monitoring of one’s cognitive processes, including knowledge of persons, tasks, and strategies (Flavell, 1979). Later models distinguished metacognitive knowledge from regulation and emphasized the reciprocal relationship between monitoring and control (Nelson, 1990; Schraw, 1998). Together, these perspectives position metacognition as the process through which learners assess their cognitive state and guide subsequent action.

This foundation is particularly relevant to GenAI-mediated learning, where learners regulate both their cognition and interaction with an external generative system. They may plan goals and prompts, monitor understanding and AI responses, evaluate accuracy and relevance, and regulate subsequent actions by revising prompts, verifying information, or accepting, modifying, or rejecting AI suggestions (Chen, 2026; Wang and Hew, 2026). The MTC Framework therefore extends established metacognitive processes to learner–AI environments, where AI outputs become objects of monitoring and inputs to subsequent regulation.

Research links GenAI use with self-regulated strategy use and metacognitive engagement across language learning (Wang and Hew, 2026). However, GenAI can both support and undermine regulation by scaffolding planning, evaluation, and reflection while also encouraging delegation of regulatory functions (Chen, 2026). EL Hosayny et al. identify planning, monitoring, revising, information management, and debugging as metacognitive processes in ChatGPT-assisted writing (El Hosayny et al., 2026). Building on this work, the present review positions metacognition as the regulatory foundation of the MTC Framework, through which learners interpret AI contributions and regulate their reliance within a broader system of trust and human–AI co-regulation.

### 2.3 Trust

Trust refers to learners’ judgement of whether an AI system’s outputs are sufficiently reliable to warrant reliance. This issue is particularly important with GenAI because fluent and plausible responses may still contain factual, linguistic, or contextually inappropriate information (Mhamdi, 2026; Li et al., 2026).

Rather than assuming that more trust is always beneficial, the present review adopts the concept of calibrated trust: learners should adjust their reliance to the reliability of a particular AI response and the demands of the task. Overtrust occurs when learners accept AI output without adequate evaluation, potentially encouraging inappropriate cognitive offloading, whereas undertrust may lead learners to disregard useful AI assistance (Abano and Estremera, 2026; Amoush and Alhosban, 2025).

Empirical studies in the reviewed corpus indicate that trust is associated with learners’ engagement, feedback acceptance, verification, and reliance on GenAI (Mhamdi, 2026; Li et al., 2026; Zhang et al., 2025). Trust is therefore not merely an attitude toward technology but a regulatory mechanism shaping AI reliance. Metacognitive monitoring provides a basis for judging whether an AI response warrants trust, while trust influences whether learners subsequently accept, verify, modify, or reject that response. Trust can thus be understood as dynamic and evidence-sensitive, linking learners’ evaluation of AI outputs to subsequent reliance decisions.

### 2.4 Human–AI co-regulation

Human–AI co-regulation extends self-regulated learning by recognizing that regulatory activity can emerge through learner–AI interaction. In GenAI-mediated learning, planning, monitoring, feedback, and decision-making may be distributed across this interaction rather than performed solely by the learner (Wang et al., 2026; Hsu, 2025). Recent work conceptualizes AI as an active participant in regulation, providing guidance, making information visible, and reshaping the conditions of self-regulated learning, while emphasizing learner control, strategic prompting, and accountability (Agustin, 2026). Thus, AI can participate in regulatory activity without becoming an autonomous learning agent.

Co-regulation is inherently interactive and iterative. AI may identify problems, generate alternatives, provide feedback, or prompt reconsideration, while learners evaluate, revise, accept, or reject these contributions (Alyasin and Shah, 2026; Alshehri et al., 2026; Huo et al., 2026). Evidence on task offloading further suggests that outcomes depend on how cognitive work is distributed, with greater benefits when AI handles selected generative functions while learners retain higher-order evaluation and regulation (Lai et al., 2026).

The present review builds on these perspectives by positioning human–AI co-regulation within a broader psychological system. In the MTC Framework, metacognition supports evaluation of AI contributions, trust shapes subsequent reliance, and co-regulation captures how that reliance is organized through interaction. The framework therefore does not introduce co-regulation as a new construct; rather, it integrates co-regulation with metacognition and trust in a theory-building account of GenAI-mediated foreign language learning.

Together, these constructs extend learning with GenAI beyond technological assistance: metacognition regulates AI-supported activity, trust shapes reliance on AI outputs, and human–AI co-regulation organizes the distribution of cognitive and regulatory work. These three constitute the MTC Framework’s core mechanisms, while AI literacy and learner agency serve as enabling capacities for informed evaluation, selective reliance, and learner control. Cognitive partnership provides the broader perspective for understanding GenAI as a cognitive resource within this regulatory system.

## 3 Methods

### 3.1 Review design

This study employed a theory-building systematic review to synthesize empirical evidence on the psychological processes underlying generative-AI-supported foreign language learning. The review followed PRISMA 2020 for study identification, screening, and reporting and used qualitative thematic and conceptual synthesis to develop a theoretical account.

The review focused on three interconnected mechanisms: metacognition, trust, and human–AI co-regulation. Related constructs, including self-regulated learning, learner agency, autonomy, AI literacy, prompt literacy, and feedback literacy, were examined as enabling conditions or associated processes. The aim was to identify recurring relationships among these constructs and explain how learners regulate their interactions with generative AI during foreign language learning.

### 3.2 Search strategy

A systematic search was conducted in the Web of Science Core Collection and Scopus for publications published between January 2022 and June 2026. The searches were conducted on 16 June 2026 in both databases. Only English-language publications were included. The databases were selected because of their broad multidisciplinary coverage of education, applied linguistics, psychology, and technology, consistent with the interdisciplinary scope of the review. Using both databases also provided complementary coverage of the literature on GenAI, foreign language education, and associated psychological processes. The core search string was:

(“generative artificial intelligence” OR “generative AI” OR ChatGPT OR “large language model*” OR LLM*) AND (“foreign language learning” OR “foreign language education” OR “second language acquisition” OR “second language learning” OR EFL OR ESL) AND (metacognit* OR “self-regulated learning” OR trust OR “AI literacy” OR “human-AI interaction” OR “human-AI collaboration” OR “human-AI co-regulation” OR “shared regulation” OR “socially shared regulation”)

Searches were conducted in titles, abstracts, and keywords where supported. Records were exported, merged, and deduplicated using EndNote. The search identified 337 records: 146 from Web of Science and 191 from Scopus. After removal of 140 duplicates, 197 records remained for screening.

### 3.3 Eligibility and study selection

Studies were included if they (1) reported original empirical research using qualitative, quantitative, or mixed-methods data; (2) examined a generative-AI or LLM-based system capable of producing natural-language outputs; (3) addressed foreign, second, or additional language learning/education; and (4) investigated at least one relevant psychological or interactional construct, including metacognition, self-regulated learning, trust, AI literacy, learner agency, autonomy, feedback literacy, motivation, human–AI interaction, collaboration, or co-regulation.

Studies were excluded if they were conceptual or non-empirical, focused exclusively on non-generative technologies, were unrelated to language learning, or examined only linguistic outcomes without addressing relevant psychological or regulatory processes.

Following PRISMA 2020, 197 records underwent title and abstract screening. Fifty-five were excluded, including 25 background-only records, 22 that did not meet the substantive inclusion criteria, and eight residual duplicates identified during screening. The remaining 142 reports were assessed at the full-text stage. Ninety-one were excluded, with reasons documented in the screening workbook. Fifty-one empirical studies met all eligibility criteria and constituted the final corpus: 25 addressing metacognition/self-regulated learning, eight addressing trust, 10 addressing human–AI co-regulation/collaboration, and eight addressing AI literacy/learner agency. The complete selection process is presented in the PRISMA 2020 flow diagram (Figure 1).

**Figure 1:**
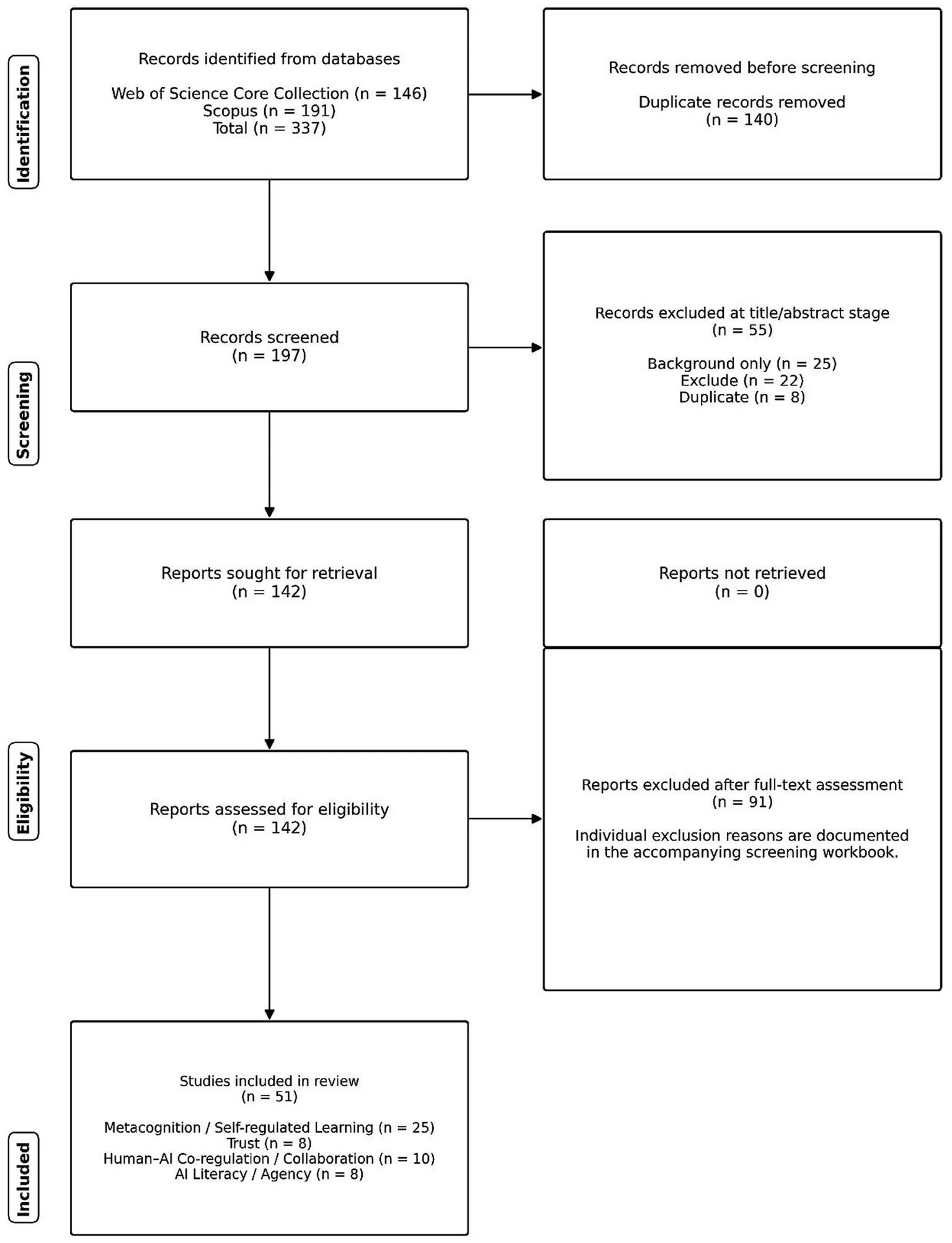
PRISMA 2020 flow diagram. source: Updated_GenAI_FLE_Screening_Workbook(2).xlsx. All PRISMA counts were verified against the corrected screening decisions.

The screening and selection process was conducted by the sole author. No second reviewer or independent coder was involved. Title/abstract screening, full-text assessment, and subsequent study selection followed the predefined eligibility criteria. Screening decisions and full-text exclusion reasons were recorded in the screening workbook to support procedural transparency.

### 3.4 Data extraction and synthesis

A structured data-extraction framework recorded bibliographic information, AI tool, language-learning context and task, participants, research design, psychological constructs and their operationalization, and principal empirical findings. The data were thematically organized around metacognition, trust, human–AI co-regulation, and AI literacy and learner agency. Data extraction and thematic coding were conducted by the sole author using the structured coding framework and four-stage synthesis procedure described below. No independent checking or inter-coder agreement procedure was used. To promote consistency, the same framework was applied across all included studies, covering the AI tool, language-learning context and task, participants, research design, psychological constructs and their operationalization, and principal findings.

The synthesis proceeded in four stages. First, findings were coded for psychological and interactional processes, including planning, monitoring, evaluation, regulation, trust calibration, AI reliance, agency, and human–AI interaction. Second, related constructs were compared across studies to identify conceptual similarities and distinctions despite terminological variation. Third, relationships among constructs were mapped, particularly how metacognitive processes informed trust and how trust and metacognitive judgment shaped subsequent AI use and regulatory behaviour. Fourth, these relationships were integrated into a theory-building framework.

The constructs were treated as interconnected rather than independent: metacognition supported the planning, monitoring, evaluation, and regulation of AI-supported activity; trust shaped reliance on AI-generated information; and human–AI co-regulation described the distribution and coordination of cognitive and regulatory activity across learner–AI interaction. AI literacy and learner agency were examined as enabling conditions that could strengthen or constrain these processes.

A formal risk-of-bias or methodological quality appraisal, such as the MMAT or CASP, was not undertaken. This decision reflected the theory-building purpose of the review and the methodological heterogeneity of the 51 included studies. Rather than applying a common quality score across diverse qualitative, quantitative, and mixed-methods designs, the synthesis focused on identifying recurring psychological and interactional relationships across studies. Nevertheless, the absence of a formal quality appraisal means that differences in methodological rigor were not systematically considered when interpreting the proposed theoretical relationships.

This synthesis informed the Metacognition–Trust–Co-Regulation (MTC) Framework, which conceptualizes GenAI-mediated learning as a recursive process in which learners plan AI-supported activity, monitor and evaluate AI outputs, calibrate trust, and regulate subsequent interaction and cognitive work. The framework is developed and interpreted in Section 5.

## 4 Results

The 51 included studies yielded four overlapping domains: metacognition/self-regulated learning (Lai et al., 2026), trust (El Hosayny et al., 2026), human–AI co-regulation/collaboration (Zakaria et al., 2025), and AI literacy/learner agency (El Hosayny et al., 2026). Because studies could address multiple constructs, these counts are overlapping contributions and should not be summed. The following sections synthesize evidence within and across these domains.

### 4.1 Metacognition and self-regulated learning

Among the 51 included studies, 25 examined metacognition and/or self-regulated learning (SRL). Collectively, these studies addressed four closely related processes in learners’ interactions with GenAI: planning, monitoring, regulation, and reflection. Although the extent and form of these processes varied across learning contexts and study designs, the evidence indicates substantial differences in how actively learners managed their engagement with AI.

Planning involved setting learning goals, identifying assistance needs, selecting strategies, and determining how to interact with GenAI. Studies of AI-supported phonetics, reading, and writing indicated that structured AI interaction could help learners organize activities and clarify task requirements (Aldamen et al., 2026a,b). Dagamseh et al. similarly showed that prompt literacy involved identifying learning needs, formulating requests, evaluating responses, and refining subsequent prompts (Dagamseh et al., 2026). Wang and Stojković found that strategic prompting enabled students to co-direct AI-supported writing and develop greater awareness of argument organization and rhetorical choices (Wang and Stojkovic, 2026). The broader SRL evidence also linked productive GenAI engagement with deliberate planning. Learners with stronger writing-SRL profiles were more likely to engage productively with GenAI, while perceived usefulness, ease of use, and interactivity were associated with GenAI use, engagement, and SRL (Liu and Zhang, 2025; Xu, 2026). Wu et al. (2026) further found that self-regulated learning strengthened the relationship between behavioral intention to use AI writing tools and writing performance. Li and Chang similarly reported gains in SRL strategies following a 16-week agentic-AI intervention, with learners’ interactions shifting from answer-seeking toward more elaborative and metacognitive questioning (Li and Chang, 2026). Jalambo et al. found that chatbot-supported self-regulated vocabulary learning was associated with increased metacognitive strategy use and learner autonomy (Jalambo et al., 2025). However, exposure to AI did not automatically result in effective planning. Stofiana et al. found that EFL learners could demonstrate metacognitive awareness while remaining relatively weak in planning and strategy adjustment, with AI use often concentrated on surface-level correction (Stofiana et al., 2025).

Monitoring concerned learners’ evaluation of the accuracy, relevance, and appropriateness of AI-generated information and feedback. Teng (2024), Teng (2025) found that stronger metacognitive awareness was associated with more critical engagement with ChatGPT feedback, whereas weaker awareness could result in uncritical copying (Teng, 2024; Teng, 2025). Bozorgian and Rahimi similarly found that although ChatGPT-4o provided consistent feedback, peer feedback more frequently encouraged self-regulatory reflection, suggesting that accurate feedback does not necessarily generate metacognitive engagement (Bozorgian and Rahimi, 2025). Learners also differed in how they evaluated AI suggestions. Guo et al. found that students incorporated some AI-generated feedback while ignoring or directly copying other suggestions (Guo et al., 2026). Lai et al. provided experimental evidence that learning was stronger when GenAI generated an initial draft but learners retained responsibility for evaluation and revision (Lai et al., 2026). Zheng and Wang likewise found that learners responded to uncertainty about GenAI through verification, reframing, selective neglect, and context-based practice (Zheng and Wang, 2026). Evidence from reading and other language-learning contexts showed similar variation in monitoring. Aldamen et al. (2026a,b) found that both AI-assisted and teacher-led explicit strategy instruction were associated with improvements in metacognitive awareness, with the AI-assisted section showing the largest descriptive gain. Ironsi and Bostanci likewise reported that structured ChatGPT-supported reading instruction was accompanied by greater learner autonomy and movement from uncritical consultation toward more selective and self-regulated use (Ironsi and Bostanci, 2026). Qiao et al. found that active GenAI use positively predicted metacognitive reading strategies through increased motivation, whereas technostress had a negative indirect relationship with these strategies (Qiao et al., 2026). Collectively, these studies show substantial variation in learners’ monitoring of AI output.

Regulation emerged when monitoring informed subsequent changes in behaviour. This included revising prompts, adapting strategies, integrating or rejecting feedback, and controlling cognitive offloading. Dagamseh et al. and Wang and Stojković documented iterative prompting in which learners inspected AI responses and refined their requests when responses were inadequate (Dagamseh et al., 2026; Wang and Stojkovic, 2026). Zhang and Jiang reported improvements in writing-SRL strategies, including planning and goal-oriented monitoring and evaluation, following LLM-supported instruction (Zhang and Jiang, 2026). Fan and Gao likewise positioned metacognitive monitoring and regulation as mechanisms associated with EFL writing outcomes (Fan and Gao, 2026). Lai et al. found stronger learning outcomes when learners retained responsibility for evaluation and revision rather than delegating these functions to AI (Lai et al., 2026). Taken together, these findings indicate that regulation involved an iterative cycle in which learners monitored AI outputs, evaluated their adequacy, and adjusted their strategies or interaction accordingly. Akbari et al. similarly found that AI-supported dynamic assessment improved writing performance and that learners’ reflections indicated a developing process of evaluating and revising AI-generated assistance within teacher-mediated instruction (Akbari et al., 2026). Other studies linked regulation to sustained engagement and strategic adjustment. Campos reported that AI-assisted feedback supported self-regulatory language learning in CLIL writing (Campos, 2025), while Zhou found that ChatGPT-supported writing was associated with improvements in perceived competence, autonomy, and self-regulatory strategies, although some learners also reported over-reliance (Zhou, 2026). Tram et al. distinguished more active and passive forms of ChatGPT-supported English learning, indicating variation in the extent to which learners actively engaged with and used the system for learning purposes (Tram et al., 2024). These findings suggest that the learning value of GenAI depended partly on whether learners used AI responses to adapt their own strategies rather than simply accept AI-generated solutions.

Reflection was examined less frequently but involved learners’ consideration of AI strengths and limitations and subsequent adaptation of their learning approaches. Aldamen et al. (2026a,b) incorporated reflective activities into AI-supported learning (Aldamen et al., 2026a,b), while Arefian and Esfandiari showed that sustained AI-supported professional learning involved repeated cycles of reflection and adaptation (Arefian and Esfandiari, 2026). At the same time, Stofiana et al. found that relatively strong monitoring and evaluation did not necessarily correspond to effective planning and strategy adjustment (Stofiana et al., 2025). Wang, Zhang, and Zhang further conceptualized GenAI-SRL as multidimensional, encompassing cognitive, metacognitive, motivational, affective, social-behavioral, and environmental regulation (Wang et al., 2026).

The 25 studies portray metacognition and SRL as dynamic and unevenly developed processes through which learners planned AI use, monitored its outputs, regulated subsequent actions, and, less consistently, reflected on the consequences of AI-mediated learning. The evidence therefore suggests that productive GenAI engagement depends not simply on learners’ exposure to AI, but on the extent to which they retain and exercise responsibility for regulating their own learning.

### 4.2 Trust in GenAI-assisted language learning

Eight studies explicitly addressed trust, trust calibration, reliability, or related judgments of AI feedback and assistance. Across these studies, trust was examined primarily in relation to trust formation, calibration, verification, reliance, and dependence, rather than as a fixed attitude toward GenAI.

Trust developed through learners’ experiences of AI’s usefulness, responsiveness, reliability, contextual fit, and pedagogical value. Tram et al. found that trust contributed to continued ChatGPT use in self-directed English learning alongside perceived usefulness and interactivity (Tram et al., 2024). Andewi et al. (2025) similarly showed that students’ willingness to rely on ChatGPT developed alongside their ability to formulate effective prompts and judge the relevance of its responses. At a broader level, Wang and Liu identified trust as an important psychological factor in GenAI adoption and highlighted perceived risks, including concerns about unreliable systems and over-reliance (Wang and Liu, 2026). These findings suggest that trust was shaped by both perceived benefits and perceived risks of GenAI use. Instructional context also shaped trust. Aldamen et al. (2026a,b) found that AI-assisted reading instruction was more productive when embedded in teacher-managed activities involving guided prompting and reflection, while Yang and Li reported that trust and perceived fairness mediated the relationship between AI-feedback quality and learner engagement (Aldamen et al., 2026a,b; Yang and Li, 2026). These findings indicate that trust was influenced not only by perceived technical performance but also by contextual and pedagogical conditions.

A prominent pattern concerned calibrated trust, whereby learners adjusted reliance according to AI reliability, task demands, contextual appropriateness, and the consequences of error. Li et al. found that clearer classroom norms, accountability, and teacher positioning of AI as contingent input were associated with more frequent verification and deeper revisions, whereas weak verification routines increased uncritical acceptance (Li et al., 2026). Alaoui Mhamdi similarly found that learners adopted, edited, verified, or rejected AI suggestions according to reliability, contextual fit, and task requirements (Mhamdi, 2026). Yin reported a comparable division of reliance (Yin, 2025). Students valued ChatGPT for speed, brainstorming, vocabulary, and other immediate assistance but consulted teachers when questions involved cultural nuance, contextual appropriateness, grading expectations, or uncertain judgments. Soyoof et al. likewise found stronger immediate and delayed learning gains from teacher-delivered than ChatGPT-delivered corrective feedback, highlighting the continuing importance of personalized and relational support (Soyoof et al., 2026).

Verification was a recurrent behavioural expression of calibrated trust. Learners checked AI-generated suggestions against teachers, other resources, or their own knowledge rather than automatically accepting them (Li et al., 2026; Yin, 2025). Trust therefore did not necessarily imply acceptance of AI output. Learners could regard GenAI as a useful source of candidate information while retaining responsibility for evaluating individual responses.

The studies also identified risks associated with overtrust and excessive dependence. Li et al. found that weak verification under assessment pressure was associated with more uncritical uptake of AI feedback and reduced revision reasoning (Li et al., 2026). Yin reported concerns that excessive ChatGPT use could weaken learners’ ownership of their writing (Yin, 2025), while Alaoui Mhamdi identified perceived dependency as a concern among graduate EFL learners (Mhamdi, 2026). Dependence thus involved not only the acceptance of inaccurate information but also the potential transfer of evaluative responsibility from the learner to AI. Conversely, learners sometimes resisted AI assistance because of concerns about inconsistency, verbosity, contextual appropriateness, or reliability (Yin, 2025). Such selective resistance occurred alongside continued use of AI for functions perceived as appropriate.

Across the reviewed studies, trust emerged as task and context-sensitive reliance rather than as a stable disposition toward GenAI. Productive reliance involved using AI when its affordances were appropriate while maintaining verification and human judgment when accuracy, contextual fit, or task consequences were uncertain. Trust thus operated in close relation to learners’ ongoing evaluation and regulation of AI use.

### 4.3 Human–AI co-regulation and collaboration

Ten studies examined human–AI co-regulation or collaboration. GenAI was used as a dialogue partner, feedback provider, cognitive scaffold, writing collaborator, and teaching assistant. Across the studies, three closely related patterns emerged: distribution of cognitive work, reciprocal interaction and shared regulation, and selective redistribution of cognitive responsibility.

The first pattern concerned the distribution of cognitive work between learners and AI. Alyasin and Shah found that students used ChatGPT across brainstorming, drafting, revision, editing, and reflection (Alyasin and Shah, 2026). Although AI helped reorganize ideas, improve clarity, and generate alternatives, students compared suggestions, rejected inappropriate outputs, and retained control over their texts. Lai et al. provided experimental evidence that the allocation of responsibilities affected learning outcomes: students performed better when GenAI generated an initial draft while they retained responsibility for evaluation and revision than when AI performed evaluative functions (Lai et al., 2026). Campos similarly found that ChatGPT provided immediate and specific feedback while learners retained responsibility for revising their writing (Campos, 2025). Li and Chang reported that agentic AI support was associated with greater autonomy, SRL, motivation, and metacognitive regulation, with interactions shifting from answer-seeking toward elaboration-seeking and metacognitive questioning (Li and Chang, 2026).

The second pattern involved reciprocal interaction and shared regulation. Several studies documented iterative exchanges in which learners modified their prompts or subsequent actions in response to AI output, while their prompts and evaluations shaped subsequent AI responses. Wang and Stojković described AI meta-prompting as cognitive scaffolding in which learners positioned AI as a critical peer or Socratic tutor and iteratively revised their prompts (Wang and Stojkovic, 2026). Alyasin and Shah similarly found that students used AI output as an intermediate object for comparing drafts, refining arguments, and reconsidering organizational choices (Alyasin and Shah, 2026). Yan showed that effective feedback seeking depended on proactive questioning and metacognitive regulation (Yan, 2025), while Yin found that learners moved between ChatGPT and teachers according to the type of assistance required, using AI for translation, vocabulary, and idea generation while consulting teachers for nuance and consequential judgments (Yin, 2025). Zhou (2026) likewise found that ChatGPT-supported L2 writing involved iterative interaction and scaffolding, although interactions focused predominantly on vocabulary and sentence-level refinement rather than broader content development.

Shared regulation was particularly evident when AI contributed to problem definition, explanation, evaluation, and revision, while learners remained responsible for interpreting and applying the resulting suggestions. Lai et al. found that students used GenAI to clarify assessment criteria, explain problems, and generate revision possibilities (Lai et al., 2026). Zhang and Jiang reported increased cognitive, metacognitive, and motivational writing strategies and more active feedback seeking, while students continued to regard teachers as important for contextualized and individualized judgment (Zhang and Jiang, 2026). Wang and Stojković similarly situated AI within teacher-designed interactional structures in which teachers established learning procedures, learners directed prompts, and AI provided dialogic scaffolding (Wang and Stojkovic, 2026). Arefian and Esfandiari found that EFL teachers repeatedly questioned, adapted, and culturally contextualized AI-generated material through cycles of individual reflection, peer discussion, and AI interaction (Arefian and Esfandiari, 2026).

The synthesis indicates that cognitive responsibility was selectively redistributed rather than completely delegated. GenAI was particularly useful for generation, explanation, brainstorming, preliminary drafting, retrieval, and immediate feedback, whereas learners retained greater responsibility for evaluation, contextual interpretation, and consequential decisions (Lai et al., 2026; Wang and Stojkovic, 2026; Zhang and Jiang, 2026; Yin, 2025). Teachers functioned primarily as instructional and contextual mediators. Thus, productive human–AI collaboration depends less on the amount of work delegated to AI than on how cognitive responsibilities are distributed between learners and AI and whether learners remain actively involved in evaluating and directing the interaction.

### 4.4 AI literacy and learner agency

Eight studies addressed AI literacy and learner agency, focusing on learners’ understanding of AI, critical evaluation of its outputs, strategic control of AI use, and maintenance of ownership and human responsibility during AI-supported learning.

The studies indicate that AI literacy extended beyond technical familiarity to include understanding AI capabilities and limitations, prompt formulation, output evaluation, verification, and ethical judgment. Lee and Jeong found that university English learners reported considerable familiarity with AI tools but weaker conceptual understanding (Lee and Jeong, 2026). Liu et al. similarly showed that Chinese EFL learners investigated how AI systems worked, compared platform affordances and limitations, and developed prompting knowledge (Liu et al., 2025). Jiang conceptualized GenAI literacy in EFL/L2 writing through understanding, intention, application, evaluation, and ethics (Jiang, 2026).

Critical evaluation was particularly evident in studies involving verification and hallucination detection. Alshehri et al. found that instruction incorporating human–AI collaboration, verification, hallucination detection, and ethical self-regulation improved academic writing and digital critical thinking (Alshehri et al., 2026). Hamamra et al. reported that learners with weaker AI literacy were more vulnerable to dependency and ethical uncertainty, whereas more critically oriented learners demonstrated more purposeful engagement (Hamamra et al., 2026). Tan found that sustained engagement was accompanied by increasingly selective judgments about when GenAI was useful and when human assistance was preferable (Tan, 2026).

Learner agency was evident when students retained responsibility for learning goals, AI-use decisions, evaluation, and ownership of their intellectual work. Alyasin and Shah found that students increasingly positioned ChatGPT as a writing partner rather than a replacement author, selectively incorporating AI suggestions while retaining control over their texts (Alyasin and Shah, 2026). Huo et al. (2026) similarly showed that learners evaluated, adapted, rejected, or sequenced teacher and GenAI feedback according to task demands and perceived credibility. Tan also reported that sustained GenAI use could coexist with continued learner ownership (Tan, 2026). Liu et al. (2025) identified variation from receptive use to participatory and co-creative engagement, reflecting differences in the extent to which learners initiated, directed, evaluated, and transformed AI-mediated activity.

The studies also highlighted a tension between AI assistance and cognitive dependence. Hamamra et al. reported that some learners recognized that ChatGPT’s efficiency could reduce their cognitive effort (Hamamra et al., 2026), whereas Alshehri et al. found that verification and ethical self-regulation could help preserve learner responsibility (Alshehri et al., 2026). Huo et al. further showed that learners differentiated feedback sources, using GenAI more readily for wording and refinement while relying more heavily on teachers for conceptual and genre-sensitive judgments (Huo et al., 2026).

A broader pattern emerges from the eight studies: AI literacy was linked to critical evaluation of AI output, whereas learner agency was reflected in strategic control over how AI assistance was used. Productive engagement involved not simply knowing how to use GenAI, but also evaluating its contributions, determining appropriate uses, and retaining ownership of consequential learning decisions.

### 4.5 Overall mapping of psychological constructs

Across the 51 studies, the psychological constructs were concentrated in four interrelated domains. Metacognition and SRL concerned learners’ planning, monitoring, regulation, and reflection; trust concerned judgments about AI reliability and appropriate reliance; human–AI co-regulation concerned the distribution and coordination of cognitive activity among learners and GenAI, often within teacher-mediated instructional contexts; and AI literacy and learner agency concerned learners’ understanding of AI, critical evaluation, and strategic control over its use. Table 1 summarizes the principal processes and representative empirical patterns identified across these domains.

**Table 1:** Principal processes and representative empirical patterns.

Psychological domain	Main processes identified	Representative evidence
Metacognition and SRL (25 studies)	Planning, monitoring, regulation, reflection	Strategic prompting, verification, feedback evaluation, revision, reflective learning
Trust (8 studies)	Trust formation, calibration, verification, reliance, dependence	Selective acceptance, source comparison, teacher consultation, calibrated reliance
Human–AI co-regulation (10 studies)	Distributed cognition, reciprocal interaction, shared regulation, cognitive redistribution	Iterative prompting, AI-supported drafting, learner evaluation and revision, teacher-mediated learner–AI interaction
AI literacy and learner agency (8 studies)	AI knowledge, critical evaluation, ownership, strategic control, human control	Hallucination detection, ethical judgment, selective AI use, differentiation of AI and teacher roles

The studies also showed substantial overlap among these domains. For example, monitoring often involved evaluating AI reliability; trust was reflected in decisions to rely on, verify, or reject AI output; and AI-supported interaction involved the redistribution of cognitive and regulatory activity. These overlaps characterize the psychological landscape identified across the reviewed studies and provide the empirical foundation for the theoretical synthesis presented in Section 5.

## 5 Discussion

### 5.1 From metacognition, trust, and co-regulation to the MTC framework

The synthesis of the 51 studies suggests that the educational value of generative AI (GenAI) depends not simply on access to the technology, but on how learners regulate their interaction with it. Building on the empirical patterns identified in Section 4, this review proposes the Metacognition–Trust–Co-Regulation (MTC) Framework of Learning with Generative AI, in which metacognition, trust, and human–AI co-regulation represent interconnected mechanisms underlying GenAI-mediated learning.

Metacognition provides the regulatory foundation for interaction with AI. The reviewed studies show that learners plan how to use GenAI, formulate and refine prompts, monitor AI-generated output, evaluate its relevance and accuracy, and reflect on the consequences of AI-supported activity. In this context, metacognition extends beyond monitoring one’s own learning to include the monitoring of an external cognitive resource whose outputs may be useful but may also be inaccurate, incomplete, or contextually inappropriate.

Trust links such evaluation to decisions about reliance. The evidence indicates that more productive learners do not simply accept or reject GenAI; rather, they adjust their reliance according to perceived reliability, task demands, contextual appropriateness, and the consequences of error. Trust is therefore conceptualized in the MTC Framework as calibrated reliance. Overtrust may encourage uncritical acceptance and excessive cognitive delegation, whereas excessive distrust may lead learners to overlook potentially useful assistance. Appropriate reliance requires learners to determine when AI output warrants acceptance, verification, modification, or rejection.

Human–AI co-regulation concerns the dynamic distribution of cognitive and regulatory activity. GenAI can support generation, reformulation, retrieval, comparison, and routine feedback, while learners retain responsibility for goals, interpretation, evaluation, verification, and consequential decisions. Human–AI synergy therefore depends on complementary and task-sensitive allocation: through prompting and evaluation, learners adjust AI involvement as task demands, uncertainty, and output quality change. Co-regulation thus enables AI to expand learners’ capabilities without displacing the cognitive activity required for learning.

The three mechanisms are best understood as recursive and mutually influencing rather than strictly sequential. Metacognitive monitoring informs judgments about AI reliability; these judgments influence the degree and form of reliance; reliance shapes how cognitive work is distributed; and the outcomes of that interaction provide new information for subsequent monitoring, trust judgments, and strategy adaptation. The resulting process can be represented conceptually as:

Metacognitive monitoring → calibrated trust → selective reliance → human–AI co-regulation → renewed monitoring and adaptation.

The arrows indicate dynamic influence rather than a fixed linear sequence. A learner may revise a prompt after detecting an inadequate response, reduce reliance after identifying an error, or increase reliance after repeated successful interactions. The MTC Framework therefore conceptualizes GenAI-assisted learning as a dynamic regulatory system in which cognition, reliance, and distributed activity continuously interact.

A further theoretical implication is the distinction between cognitive augmentation and cognitive substitution. The critical issue is not the amount of AI involvement but whether its use preserves learners’ engagement in the higher-order processes through which learning develops. Productive augmentation occurs when AI assumes selected processing functions while learners retain responsibility for planning, reasoning, evaluation, and reflection; substitution occurs when these core learning functions are delegated to AI. This distinction provides a basis for understanding when AI-supported activity enhances rather than displaces learning.

### 5.2 Theoretical interpretation

The MTC Framework extends self-regulated learning (SRL) perspectives, but its contribution is not simply to apply established metacognitive or SRL concepts to GenAI. Metacognition, trust, and co-regulation are each established constructs. The theoretical contribution of the present framework lies in integrating them to explain how learners regulate cognitive responsibility during interaction with an interactive generative system.

First, the framework positions calibrated trust as a link between metacognitive evaluation and reliance. Learners must not only evaluate their own learning and AI-generated information; they must also judge whether an AI output warrants acceptance, verification, modification, or rejection. Trust therefore helps explain variation in learners’ reliance on similar AI outputs across tasks and contexts.

Second, the framework conceptualizes cognitive responsibility as dynamically distributed rather than simply transferred. GenAI may undertake selected functions such as generation, reformulation, retrieval, brainstorming, or routine feedback, while learners retain responsibility for goals, interpretation, evaluation, verification, and consequential decisions. The issue is therefore not whether cognitive work is shared with AI, but whether its distribution preserves the cognitive activity required for learning.

The recursive integration described in Section 5.1 constitutes the framework’s central theoretical proposition. Metacognitive monitoring may inform judgments of AI reliability, trust may shape selective reliance, and reliance may influence the distribution of cognitive responsibility, with subsequent interaction providing information for renewed monitoring and adaptation. This recursive relationship is a theory-building integration of recurring patterns across the reviewed studies rather than a causal relationship directly established by the evidence.

AI literacy and learner agency function as enabling capacities rather than additional MTC mechanisms. They support learners’ understanding and critical evaluation of AI, informed reliance, and retention of control over AI-supported activity.

Overall, the MTC Framework extends rather than replaces SRL by specifying how established regulatory processes may become interconnected when cognitive activity is distributed through interaction with a generative system. Its contribution is therefore to provide a psychological account of how metacognition, calibrated trust, and human–AI co-regulation jointly shape the organization of cognitive responsibility in GenAI-supported learning.

### 5.3 Conditions for effective GenAI-assisted learning

The MTC Framework suggests that effective GenAI-assisted learning requires instructional arrangements that keep learners cognitively engaged throughout the interaction. AI-supported activities should therefore involve processes such as planning, evaluation, verification, revision, and reflection rather than merely retrieving completed answers. AI-generated output is most productive when it becomes material for further learner judgment and action.

Effective use also requires tasks that make evaluation and verification part of the learning process. Learners can be encouraged to compare AI responses with other sources, identify limitations, justify the acceptance or rejection of suggestions, and revise their work accordingly. Such practices can help transform AI interaction from answer retrieval into an opportunity for learning and reduce passive acceptance of AI-generated content.

The distribution of cognitive work should also be considered in task design. Task design should therefore assign AI-supported functions deliberately and reserve core learning processes for learners. For example, AI may generate alternatives or preliminary material, while learners compare options, justify selections, revise outputs, and reflect on the resulting learning.

Finally, the evidence indicates that teacher mediation remains important. Teachers can establish learning goals and interactional structures, contextualize AI-generated feedback, and support learners in evaluating and appropriately using AI contributions. They can also help learners determine when AI assistance is appropriate and when independent cognitive engagement should take priority. Effective GenAI-assisted learning is therefore better characterized as human-directed co-regulation than as autonomous AI-mediated learning.

### 5.4 Summary of findings and answers to the research questions

The five research questions are addressed by integrating the empirical evidence synthesized in Section 4 with the theoretical interpretation developed in Sections 5.1–5.3 (Table 2).

**Table 2:** Summary of findings.

Research question	Answer from the review
*RQ1*. What psychological processes and constructs have been investigated?	Across the 51 studies, the most prominent constructs were metacognition/SRL, trust, human–AI co-regulation, AI literacy, and learner agency. Related constructs included autonomy, motivation, engagement, self-efficacy, feedback literacy, and cognitive load.
*RQ2*. How does metacognition shape engagement with GenAI?	Metacognition shapes how learners plan AI use, monitor and evaluate AI output, regulate prompts and strategies, and reflect on learning. Productive engagement was associated with learners retaining responsibility for evaluation and regulation rather than accepting AI output passively.
*RQ3*. How do learners develop and calibrate trust?	Trust was shaped by perceptions and experiences of AI’s usefulness, reliability, responsiveness, and contextual appropriateness. Productive reliance involved verification, selective acceptance, modification, rejection, and consultation with teachers rather than unconditional acceptance of AI output.
*RQ4*. How do learners and AI participate in human–AI co-regulation?	Co-regulation involved distributed cognition, reciprocal interaction, shared regulation, and selective redistribution of cognitive work. AI commonly supported generation, explanation, drafting, and feedback, while learners and teachers retained greater responsibility for goals, evaluation, contextual judgment, and consequential decisions. AI literacy and learner agency supported strategic management of this distribution.
*RQ5*. How can these relationships be integrated theoretically?	The evidence supports the MTC Framework, in which metacognition regulates learner–AI interaction, trust calibrates reliance, and co-regulation organizes the distribution of cognitive and regulatory work. AI literacy and learner agency function as enabling conditions for these processes. The framework proposes that learning is most productive when GenAI augments rather than substitutes for learner cognition.

The synthesis indicates that the educational value of GenAI lies not in maximizing AI involvement but in establishing a regulated human–AI relationship in which GenAI extends cognitive resources while learners retain epistemic agency, critical judgment, and responsibility for learning.

### 5.5 Implications

The findings have implications for theory and pedagogy. Theoretically, the MTC Framework extends SRL to learning environments in which cognitive activity is distributed between learners and generative systems. It shifts attention from whether GenAI is effective per se to how metacognition, calibrated trust, and co-regulation shape learning. From an educational-psychology perspective, it conceptualizes AI-supported learning as distributed self-regulation, in which learners monitor their cognition while regulating reliance on an external generative system. Trust calibration thus functions as a dynamic mechanism linking metacognitive judgments to reliance decisions.

Pedagogically, GenAI instruction should move beyond operational proficiency toward learning-oriented use by explicitly mapping activities onto MTC processes. Prompt planning supports metacognitive planning, while output checking and comparison of alternatives develop monitoring of AI-generated content. Reliability judgments support trust calibration by requiring learners to assess when AI output is accurate and contextually appropriate. Accept–modify–reject decisions develop reliance regulation while preserving learner responsibility for consequential judgments. Reflective evaluation links these processes by prompting learners to reconsider decisions and adapt subsequent AI use.

For teachers, this implies designing tasks in which AI complements rather than replaces learner cognition, while learners retain responsibility for consequential judgments. For learners, it highlights questioning, verifying, modifying, and, when necessary, rejecting AI-generated output. At the curriculum and institutional levels, the findings support integrating AI literacy with metacognitive and feedback literacy rather than treating AI competence as purely technical.

### 5.6 Limitations and future research

Several limitations qualify the interpretation of the findings. The 51 studies varied substantially in participants, research designs, learning contexts, AI applications, and operationalization’s of psychological constructs, which limits direct comparison across studies. Moreover, the evidence base is relatively recent and rapidly evolving; findings associated with particular GenAI systems may therefore change as technologies and patterns of use develop.

The review has methodological limitations related to its single-author design. Screening, full-text assessment, and thematic coding were conducted by the sole author without independent reviewer or coder verification, which may have introduced selection or interpretive bias despite the use of a structured coding framework and documented screening decisions. No formal risk-of-bias appraisal was conducted, reflecting the theory-building focus and methodological heterogeneity of the included studies. The database coverage also represents a limitation. Although Web of Science and Scopus provided broad multidisciplinary coverage, PsycINFO and ERIC were not searched. Future reviews should incorporate education and psychology-specific databases to broaden coverage and reduce potential database-related selection bias. Overall, the MTC Framework should be viewed as a theory-generating account that requires further empirical validation.

The evidence also relied substantially on self-reports, interviews, surveys, and relatively short-term interventions. Although these approaches provide valuable insights into learners’ perceptions and experiences, they may not fully capture how metacognitive regulation, trust calibration, and cognitive redistribution unfold during actual learner–AI interaction. Future research should therefore incorporate more longitudinal and process-sensitive approaches, including interaction logs, prompt histories, revision records, think-aloud protocols, and screen-based observation.

Further research should examine how learner proficiency, prior AI experience, AI literacy, metacognitive ability, task complexity, instructional design, and teacher mediation shape MTC processes. In particular, the proposed framework requires empirical testing. Longitudinal, experimental, and mixed-methods research could examine whether metacognitive engagement is associated with trust calibration, whether calibrated reliance is associated with particular forms of co-regulation, and whether these processes are subsequently related to language-learning and psychological outcomes.

## 6 Conclusion

This systematic review examined the psychological processes through which learners engage with generative artificial intelligence (GenAI) in foreign language education. Drawing on 51 empirical studies, it moved beyond whether GenAI improves particular language skills to examine how learners understand, evaluate, regulate, and coordinate their interactions with generative systems. Metacognition, trust, and human–AI co-regulation emerged as interconnected mechanisms underlying GenAI-mediated learning, while AI literacy and learner agency functioned as enabling conditions.

The findings conceptualize learning with GenAI as adaptive human–AI partnership. Metacognition supports goal setting, monitoring of AI contributions, evaluation, and subsequent regulation. Trust calibration shapes decisions about whether AI-generated information or feedback should be accepted, verified, modified, or rejected. Human–AI co-regulation captures the distribution and coordination of cognitive and regulatory work between learners and AI. These mechanisms are neither discrete nor strictly sequential; rather, they form a dynamic system in which metacognitive evaluation informs trust and reliance, reliance shapes cognitive distribution, and interaction outcomes provide new information for subsequent regulation.

The educational value of GenAI therefore depends less on its capacity to generate language, provide feedback, or perform cognitive tasks than on how learners incorporate these affordances into their regulatory activity. GenAI can augment learning when learners retain responsibility for planning, evaluation, judgment, and reflection. When these functions are increasingly delegated to AI, assistance may become cognitive substitution, potentially weakening independent engagement and learner control. Productive augmentation and problematic dependence thus depend primarily on how cognitive and regulatory responsibility is organized.

The Metacognition–Trust–Co-Regulation (MTC) Framework integrates these processes into a unified theoretical account. It explains how cognitive activity can be distributed between learners and AI while preserving human agency as the organizing principle of learning. AI literacy and learner agency are conceptualized not as additional MTC mechanisms but as enabling conditions that help learners understand GenAI’s capabilities and limitations, calibrate reliance, and retain ownership of learning. The framework therefore provides a basis for explaining variation in psychological and learning outcomes, including autonomy, motivation, confidence, engagement, cognitive load, feedback literacy, and agency.

The principal contribution of this review is to specify the psychological conditions under which GenAI becomes educationally productive, rather than characterizing it as inherently beneficial or detrimental. This perspective shifts attention from evaluating GenAI primarily as an instructional technology toward understanding how learners regulate its use while preserving the cognitive activities through which learning occurs.

Future research should empirically test the MTC Framework across learner populations, language tasks, educational settings, and GenAI systems. Longitudinal, experimental, and process-sensitive designs are needed to examine whether the proposed relationships among metacognition, trust, and co-regulation remain robust across contexts and over time. Research should also identify when human–AI cognitive partnerships enhance learning without displacing essential cognitive and regulatory activities. The central challenge for GenAI-enhanced foreign language education is not how much learning can be delegated to AI, but how AI can be integrated while preserving and strengthening learners’ capacity to think, evaluate, regulate, and act independently.
